# Care Burden and Coping Strategies among Caregivers of Paediatric HIV/AIDS in Northern Uganda: A Cross-Sectional Mixed-Method Study

**DOI:** 10.1155/2021/6660337

**Published:** 2021-09-13

**Authors:** Ibrahim Mujjuzi, Paul Mutegeki, Sarah Nabuwufu, Ashim Wosukira, Fazirah Namata, Patience Alayo, Sharon Bright Amanya, Richard Nyeko

**Affiliations:** ^1^Lira University, P.O. Box 1035, Lira, Uganda; ^2^Department of Microbiology and Immunology, Lira University, P.O. Box 1035, Lira, Uganda; ^3^Department of Paediatrics and Child Health, Lira University, P.O. Box 1035, Lira, Uganda

## Abstract

**Background:**

Family caregivers provide the bulk of care to children living with HIV. This places an enormous demand and care burden on the caregivers who often struggle to cope in various ways, some of which may be maladaptive. This may adversely affect their quality of care. Very little literature exists in resource-limited contexts on the burden of care experienced by caregivers on whom children living with HIV/AIDS depend for their long-term care. We assessed care burden and coping strategies among the caregivers of paediatric HIV/AIDS patients in Lira district, northern Uganda.

**Methods:**

A mixed-method cross-sectional study was conducted among 113 caregivers of paediatric HIV patients attending the ART clinic at a tertiary healthcare facility in Lira district, northern Uganda. A consecutive sampling method was used to select participants for the quantitative study, while 15 respondents were purposively sampled for the qualitative data. Quantitative data were collected using standard interviewer-administered questionnaires, while in-depth interview guides were used to collect qualitative data. Data were entered, cleaned, and analysed using SPSS version 23. Qualitative data were analysed thematically.

**Results:**

The majority of the caregivers, 65.5% (74), experienced mild-to-moderate burden. The mean burden scores significantly differed by caregivers' age (*P*=0.017), marital status (*P*=0.017), average monthly income (*P*=0.035), and child's school attendance (*P*=0.039). Accepting social support, seeking spiritual support, and reframing were the three most commonly used strategies for coping. Marital status and occupation were, respectively, positively and negatively correlated with information-seeking as a coping strategy, while monthly income was positively correlated with psychosocial support as a strategy. Seeking community support was negatively correlated with the duration of the child's care.

**Conclusions:**

Our findings show that care burden is a common problem among the caregivers of children living with HIV in the study context.

## 1. Introduction

It is estimated that about 1.7 million of the over 37.9 million people living with HIV globally in 2018 were children aged below 15 years, the majority of whom are in sub-Saharan Africa [1]. In Uganda, the country with the fifth-highest prevalence in the region, up to 100,000 (7.1%) of the estimated 1.4 million people living with HIV in 2018 were children below the age of 15 years, of which an estimated 7,500 were new HIV infections [2]. The high burden of HIV has resulted in both direct and indirect effects on the population in low- and middle-income countries, leading to various social and economic challenges for an already vulnerable group of people [3]. Although antiretroviral drugs and treatments have burgeoned, the burden of caregiving has not changed [4]. Antiretroviral therapy (ART) has reduced morbidity and mortality among people living with HIV, including children, thus making HIV become a chronic disease [5]. Chronic diseases, and therefore HIV, not only affect the lives of those suffering from the illness but also affect the lives of family members who take care of them [6], with both positive and negative consequences. Providing chronic care to children living with HIV/AIDS presents unique demands and burdens to families and the entire healthcare system [7], often associated with negative effects on caregivers [4, 8]. The negative effect of caregiving has been described as a caregiver burden [9] and it encompasses the physical, social, emotional, and financial toll of providing care [4, 8]. According to Chandran et al., caregiver burden refers to “the physical, emotional, and financial hardships associated with providing care to a diseased individual” [5]. In low resource settings, Uganda inclusive, care burden is often contributed to by high levels of poverty, illiteracy, and disruption of family social support systems. Pieces of evidence suggest an increasing level of stress in caregiving and this requires adequate attention to understand and help reduce this stress [4, 10].

Caregivers and, by extension, families have often struggled to cope with this burden through various ways, including concealment of the child's health status, drawing strength from their faith and belief in God, and reaching out for support. These reactions to cope with stressful situations and the demands of caregiving can be adaptive or maladaptive, where some caregivers adapt well, while others do not [11, 12]. Failure by caregivers of children living with HIV to appropriately cope can adversely affect care-related outcomes, including poor retention in chronic HIV care and poor adherence to ART, consequently contributing to low viral load suppression among children.

While several studies have been done to assess care burden and identify coping strategies among caregivers, these have majorly centered on adults and other debilitating diseases such as psychiatric disorders, dementia, autism, and general HIV patients [13–18]. Little attention has been paid to the care burden experienced by persons on whom children living with HIV depend for onward lifelong support, and there is a paucity of data on the same in resource-limited contexts like the current study setting. The few studies that focused on caregivers of children were largely qualitative and in contexts that differ from that of the current study setting [4]. This study, therefore, assessed care burden and coping strategies among caregivers of paediatric HIV/AIDS patients in a resource-poor setting in northern Uganda.

## 2. Methods

### 2.1. Study Design and Setting

We used a cross-sectional mixed-method design to collect quantitative and qualitative data during August 2020. The study was conducted in the antiretroviral therapy (ART) clinic of Lira Regional Referral Hospital (LRRH), a tertiary care health facility in Lira district, northern Uganda. The facility receives patients from over 9 districts in the subregion and beyond, with a catchment population of about 2.3 million, offering a wide range of general and specialized curative, promotive, and preventive health services. To date, the facility has over 34,000 clients enrolled on ART, about 500 of whom are children under 15 years. This site was selected because of its high client load, in addition to serving clients referred from all ART clinics in the subregion, and therefore provides a relatively good representative population. Paediatric HIV services in Uganda and the study context are provided according to the national ART guidelines, revised in December 2016 to include initiating all HIV-infected clients on ART regardless of age, clinical stage, and CD4 cell count, the “test and treat” policy [19]. The HIV services are provided free with support from PEPFAR funding and include but are not limited to HIV testing services, ART, prophylaxis for opportunistic infections, adherence counselling and psychosocial supports, and routine clinical and laboratory monitoring. Paediatric HIV services are largely facility-based, especially for younger children who are followed up according to the national guidelines with an evaluation at 2 weeks after initiation of ART, every month for the next several months, and every 3 months afterwards. During the follow-up visits, standard medical care is provided to all persons on ART routinely or as and when required, including counselling, pick-up of prescriptions (antiretroviral drugs, cotrimoxazole, and other drugs), physician evaluations, and laboratory testing (CD4 lymphocyte count and viral load).

### 2.2. Study Population

Our study comprised caregivers of paediatric HIV/AIDS patients aged 2–12 years who received ART services from a tertiary level facility and who have spent at least 6 months caring for the child. The caregivers were drawn from the HIV care clinic at the study site as they came in for their ART appointments. In the study context, caregivers of paediatric HIV, on whom the children depend for most of their support, comprise a mixed group of individuals but mainly the biological mothers of the children who are themselves HIV-infected and receiving HIV care and treatment. A significant number of HIV-infected children are also cared for by either a sibling or other close relatives, occasioned by the high number of orphans resulting from the over two decades of insurgency in northern Uganda and the HIV scourge itself.

### 2.3. Sample Size Estimation

#### 2.3.1. Quantitative Data

The method for estimating the sample size for cross-sectional studies [20] was used for this study, based on the following formula: *N* = *Z*^2^*p* (1 − *P*)/*e*^2^, at 95% level of confidence, with *P*=8% [21] and allowable error (*e*) of 5%. The estimated sample size of 113 was obtained.

#### 2.3.2. Qualitative Data

We interviewed 15 respondents for the qualitative data. These were conveniently selected for in-depth interviews because of their depth of experience in caring for HIV-infected children, a process that was carried on until saturation was reached.

### 2.4. Sampling Criteria

We used a consecutive sampling technique to recruit study participants for quantitative data, while a purposive sampling technique was used to select respondents for the individual in-depth interviews.

### 2.5. Data Collection Instruments

For quantitative data, we used a standard 22-item Zarit Burden Interview (ZBI) tool [22] to assess caregivers' perceived burden of providing care. The questions focus on the caregiver's health, psychological well-being, finance, social life, and interpersonal relationships that cause stress and strain. The reliability of the ZBI tool measured by Cronbach's coefficient has been reported to range from 0.77 to 0.94 [11, 22–24]. The 22 items are assessed on a 5-point Likert scale, ranging from 0 = “never” to 4 = “nearly always.” Individual item scores are added up to give a total score ranging from 0 to 88, with higher scores indicating a higher perceived burden. The cut-off points for the ZBI were as follows: 0–20 (little or no burden), 21–40 (mild-to-moderate burden), 41–60 (moderate-to-severe burden), and 61–88 (severe burden). Besides, a standard 29-item Family Crisis Oriented Personal Evaluation Scale (F-COPES) was used to assess caregivers' coping. The F-COPES has an internal consistency of 0.89 [15, 25] and is based on a 5-point scale with scores ranging from 1 to 5, where 1 = strongly disagree, 2 = moderately disagree, 3 = neither agree nor disagree, 4 = moderately agree, and 5 = strongly agree. The five subscales designed in the F-COPES include acquiring social support, reframing, seeking spiritual support, mobilizing the family to acquire and accept help, and passive appraisal [25]. The tools were used to collect sociodemographic information, care burden, and coping strategies.

For the qualitative data, we developed an in-depth interview guide in line with our study objectives to explore caregivers' perspectives and experiences of caring for HIV-infected children.

### 2.6. Data Collection

#### 2.6.1. Quantitative Data

Caregivers of paediatric HIV clients who accessed ART services from LRRH were identified at the time of their appointment visit. Data were collected from consenting participants using an interviewer-administered questionnaire after explaining the purpose, research procedure, and their rights as participants in the study. The interview took approximately 20–25 minutes.

#### 2.6.2. Qualitative Data

Individuals identified for the qualitative study were approached and those who agreed to participate were interviewed after giving informed consent. The interview was conducted in a convenient private room within the ART clinic to explore the care burden and coping approaches of the caregivers. It was moderated by the researchers using a semistructured in-depth interview guide and audio-recorded, in addition to taking notes. Each session took about 30 minutes and was conducted in the local language which was best understood by the respondents. Questions on the in-depth interview guide included the respondent's demographics, relationship with the child, experiences in caring for a child with HIV, burdens or difficulties faced in caring for the child, ways of coping with the burden, and general views on caring for HIV-infected children. Probes depended on a respondent's experiences and clarity of their narratives. The interviews were later translated and transcribed into the English language.

### 2.7. Data Management and Analysis

#### 2.7.1. Quantitative Data

Completeness of data was ensured during data collection through daily reviews and taking corrective actions. Data were entered, cleaned, and analysed using Statistical Package for Social Sciences (SPSS) software (IBM SPSS Statistics for Windows, Version 23.0, Armonk, NY: IBM Corp.). Descriptive statistics were used to summarize the data obtained from the participants. Continuous variables with approximately normal distribution were described using means (standard deviations), while no normally distributed variables were described using medians (interquartile ranges). The analysis of variance (ANOVA) and independent *t*-tests were used to examine the differences in the mean burden scores regarding sociodemographic characteristics. An exploratory factor analysis using principal component analysis with varimax rotation was used on the coping data to assess the empirical support of the original scales applied to this sample of the population. Factor analysis provides a preliminary analysis of how a scale measures the concepts it is designed to measure [13]. The scree test and the eigenvalues >1 rule and a factor loading of at least 0.35 [26] were used to determine the number of factors. Factors with at least three items loaded on them were viewed as more psychometrically stable [27]. Internal consistency was estimated using Cronbach's *α* coefficient. Pearson correlation coefficient and multiple linear regression analyses were used to assess the relationship between the coping scales and sociodemographic characteristics. Statistical significance was set at *P* < 0.05.

#### 2.7.2. Qualitative Data

The qualitative data generated from the in-depth interviews were transcribed and analysed manually using content thematic analysis. The researchers read, coded, and agreed on the subthemes. The analysis focused on the responses around the experiences and challenges of providing caregiving and the strategies often adopted by the respondents in trying to overcome these challenges or burdens of caregiving. This first step in the analysis was aimed at carefully reading the transcripts and noting down initial views about each participant, a within-case analysis in each transcript and noting themes. The next step involved comparing the themes from one case to the other across all the transcripts and noting themes that were relevant to the research questions. Ultimately, relevant compelling quotes which represented lucid elements of our working themes were selected.

## 3. Results

### 3.1. Quantitative Results

#### 3.1.1. Sociodemographic Characteristics of Study Participants

The majority, 75.2% (85), of the 113 respondents were females, with a median age of 38 years (IQR 12) and an age range of 18–74 years. More than one-half of the caregivers, 69.0% (78), were biological parents of the child, while 31.0% (35) were other relations that typically comprised other extended family members (Table 1). At least 16.8% (19) of the respondents were caring for two or more children living with HIV. The median age of the children was 9 years (IQR 4), over half of whom were females (55.8%), and at least 15.0% (17) had been in care for over 10 years (median 5 years [IQR 4]). Up to 21.2% (24) of the children had been generally sickly despite ART. The rest of the sociodemographic characteristics are as shown in Table 1.

#### 3.1.2. Descriptive Statistics (Mean/SD) of Individual Care Burden Scores

Table 2 summarizes the individual mean care burden scores related to each of the 22-item ZBI care burden questions. The mean scores for the individual questions ranged from a low of 0.53 for the question “Do you feel uncomfortable about having friends because of your child?” to a high of 3.58 for the question “Do you feel you should be doing more for your child?” Moreover notable was the low mean score relating to the question “Do you feel that you don't have enough money to take care of your child in addition to the rest of your expenses?” (mean = 1.23). Similarly, the mean score on the question “Do you feel that your child negatively affects your relationships with other family members or friends?” was low (mean = 0.89). The low ranking of the question “Do you feel you have lost control of your life since your child's illness? (mean = 0.64) demonstrates a type of resilience and shows that the “burn-out syndrome” has not yet cropped up among the caregivers in the study context (Table 2).

#### 3.1.3. Level of Care Burden

**Figure 1:**
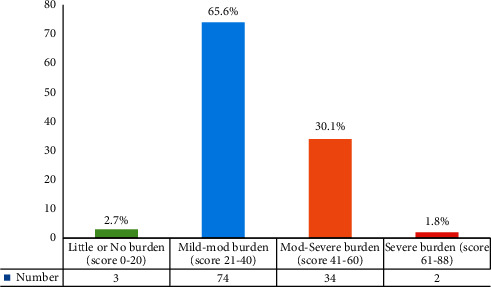
Level of care burden experienced by caregivers.

The overall mean burden score was 36.9 ± 9.7, where the respondents experienced one form of care burden or the other to varying extents. The majority of the caregivers, 65.6% (74), had mild-to-moderate care burden, 30.1% (34) had a moderate-to-severe burden, 2.7% (3) had little or no burden, and only 1.8% (2) had severe burden (Figure 1).

#### 3.1.4. The Relation between Care Burden and Caregivers' Sociodemographic Characteristics

The mean care burden score was significantly higher among caregivers in the age range of 30–39 years (39.07 ± 10.70, *P*=0.017), caregivers who were divorced (44.00 ± 14.46, *P*=0.017), and those with an average monthly household income less than $67 or approximately $2 a day (39.24 ± 10.14, *P*=0.035) (Table 3). Likewise, respondents caring for HIV positive children who were attending school had lower burden scores (36.30 ± 9.30) compared to those caring for children who were not in school (42.64 ± 11.99), and this difference was statistically significant, *P*=0.039. The burden scores relating to the rest of the caregiver and child sociodemographic characteristics are as shown in Table 3.

#### 3.1.5. Descriptive Statistics of Coping Strategies Assessment

Table 4 summarizes the mean scores for each item in the 29-item F-COPES used to assess coping strategies among the respondents. This ranged from a low of 1.77 for the strategy relating to item C26 “seeking advice from a minister” to a high of 4.99 relating to item C29 “having faith in God” as a coping strategy. A mean score per item of greater than 3.0 indicated that the item was a support component strongly used by the respondents (Table 4), implying, therefore, that there was a good range of coping options adopted by the caregivers who participated in this study, given that 22 out of 29 options had an individual mean score of ≥3.0.

**Figure 2:**
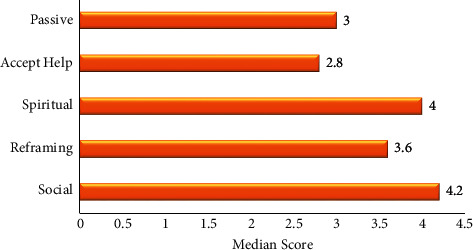
Extent of caregivers' usage of the F-COPES subscale support system.

Furthermore, as shown in Figure 2, the respondents used the original five subscales as coping strategies to varying extents. Acquiring social support was the strongest support system and ranked highest (median = 4.2) in the extent of use, followed by seeking spiritual support (median = 4.0) as the next highly ranked support system, while acquiring and accepting help (median = 2.8) was the weakest coping strategy (Figure 2). The internal consistency of the scale for this study, as indicated by the Cronbach's alpha estimate, was 0.68, while the interitem coefficients (Cronbach's *α*) for each of the original five subscales were Acquiring Social Support (0.69), Reframing (0.74), Seeking Spiritual Support (0.09), Acquire & Accept Help (0.40), and Passive Appraisal (0.48).

#### 3.1.6. Relation of Coping Strategies with Caregivers' Sociodemographic Characteristics

A principal component factor analysis with varimax rotation was performed on the coping data to assess the experiential support for the original five scales applied to the current sample population. Using an eigenvalue of >1.0 as the criterion resulted in 10 factors being extracted from the entire pool of items. However, based on the original five-factor scale and given the marked drop in the percentage of variance explained by the sixth factor on the scree plot, a five-factor solution was accepted as the best one and was used for the exploratory factor analysis. These explained 47.3% of the total variance in the 29-item F-COPES as applied to the current study population. The new explanatory factors could be categorized as follows: Factor 1: Internal Strength (6 items: 3, 7, 11, 12, 13, and 21), accounting for 13.9% of the variance; Factor 2: Seeking Community Support (4 items: 8, 10, 27, and 28), accounting for 11.6% of the variance; Factor 3: Information Seeking (7 items: 1, 2, 4, 5, 16, 19, and 24), accounting for 8.9% of the variance; Factor 4: Acceptance (4 items: 15, 22, 23, and 25), accounting for 6.9% of the variance; and Factor 5: Psychosocial Support (4 items: 6, 9, 20, and 26), accounting for 6.0% of the variance. Each item's loading on the five extracted factors is shown in Table 5. The Cronbach's alpha estimates for the five extracted factors were higher than those for the original subscales and ranged from 0.51 for the Psychosocial Support subscale to 0.80 for the Internal Strength subscale. The overall Cronbach's *α* coefficient for the factors loaded (25 items) was 0.71. Four items of the original 29-item F-COPES “dropped out” by not loading on any factor greater than 0.35. These were “attending church services” (item 14), “knowing luck plays a big part in how well we can solve family problems” (item 17), “accepting that difficulties occur unexpectedly” (item 18), and “having faith in God” (item 29).

#### 3.1.7. The Relationship between Coping Strategies and Sociodemographic Characteristics

Table 6 shows the correlation and multivariate linear regression beta coefficients of the relationship between the extracted coping subscales and respondents' sociodemographic characteristics. Marital status was positively correlated with Information Seeking, implying that the use of information-seeking becomes more frequent as the marital status changes from “single” to “widowed.” Likewise, the level of monthly income showed a positive relationship with the Psychosocial Support subscale, indicating that caregivers with higher income used more psychosocial support for coping. There was a negative correlation between occupation and Information Seeking, implying that caregivers less frequently used information-seeking strategies as their occupation status tends towards being unemployed. Caregivers used less of the strategy of Seeking Community Support the longer the child remained in care.

### 3.2. Qualitative Results

#### 3.2.1. Care Burden

In this study, we sought to address two major research questions: care burden and the coping strategies among caregivers of paediatric HIV/AIDS patients. From the qualitative analysis, the following themes emerged as having major bearings on care burden: financial, psychosocial, health facility-related, and child health-related burdens.

*(1) Financial Burden*. Financial constraints were variously expressed as a key burden by nearly all the caregivers. This is related mainly to the inability to provide for the basic family and the child's needs in terms of feeding, clothing, shelter, and education, often forcing the caregivers to do casual labour, described as “odd jobs,” to sustain the family needs. This meant that meeting the family and the child's other needs would sometimes take priority over the visits to the health facility for the child's related medications. Caregivers lacked the time and the means of meeting transport costs to take their children to the clinic. This was a major barrier as exemplified by statements from some of the respondents who asserted the following:

“*...it is hard taking care of him daily because he would love to eat nice things like meat, fish. Sometimes he sees those nice things from neighbours and it is usually not affordable for me...I also have to work hard weeding people's gardens to take care of the children and sometimes I am not paid on time*,” said a 54-year-old grandmother.

“*...at times, she refuses to take her medication because I have not been able to afford to buy for her good food such as “mukene”(silverfish) and others*...,” said a 29-year-old mother.

“*...I do odd jobs to take care of the child and as I am old, it is hard for me*,” said a 44-year-old grandmother.

*(2) Psychosocial Burden.* Most responses related to psychosocial burden rotated around stigmatization, discrimination, and disclosure of the child's status. Caregivers expressed being stigmatized and discriminated against because of their child's HIV status, mainly from within the community where they live. For some caregivers, this hindered their ability to care for the child effectively, since they could not give their children the drugs in the presence of their neighbours and friends.

A 36-year-old caregiver taking care of an orphaned HIV-infected child said:

“...*the neighbours also fear me because they think I also have HIV/AIDS. I also had fears that caring for this child will lead me into contracting it (HIV/AIDS).*”

Disclosure of the child's and/or the caregiver's HIV status was another challenge expressed by the caregivers as presenting a huge psychological burden, since the children have often questioned why they needed to take the drugs daily while other children/siblings did not. Lack of disclosure also resulted in various hurdles in caregiving, including poor adherence:

“*...it was also hard telling the child that he is positive and it is hard making him adhere to his drugs since he doesn't see his friends taking drugs,”* asserted a 57-year-old male caregiver.

Also related was the effect of parenting on care burden, which was highlighted by some respondents as exemplified by the following statement:

“...*the most painful of my experiences was the loss of this child's mother. From then, I found it very difficult to give the medication to the child, defaulting medication time*...,” said a 36-year-old female caregiver of an orphaned HIV-infected child.

*(3) Healthcare-Related Burden.* Caregivers highlighted various healthcare-related components as factors contributing to deterrent and burden in caring for the HIV-infected child, especially as far as health facility appointment visits were concerned. The long waiting hours in the health facility on appointment dates and the clashing of the child's and the caregiver's appointment dates for some caregivers who are receiving care from different health facilities were reported to be too burdensome, in addition to the prohibitive transport costs. The following quotes exemplify such concerns:

*“…the child picks drugs here from Lira regional referral and sometimes the dates for picking his drugs clashes with mine in Otuke health centre,”* said a 57-year-old HIV-positive caregiver.

*“...lack of transport money to come to the clinic on appointment date sometimes make us miss picking drugs on time and the child misses some doses,”* commented a 37-year-old mother.

*(4) Child's Health-Related Burden.* For some caregivers, constant sicknesses of the HIV-infected children under their care were reported to be challenging, since this necessitated frequent visits to the health facilities for medications:

“*I have been suffering from nursing him in the hospital considering that he is always ill especially when he had TB...,*” stated a 37-year-old mother.

“*....I am a widow and now acting as a mother as well as a father. Therefore, I always find it very difficult to give proper care to this child and other children as well... getting what to eat is always a challenge and at times whenever this child is admitted to the hospital, we stay there hungry since we lack what to eat*,” lamented a 41-year-old mother of six.

Medication time was reported by most of the caregivers as a challenging and burdensome task, given their fixed schedules which should be administered or supervised by the caregivers, especially for younger children. This was reported to result, sometimes, in the medication being administered to the children late. Likewise, defaulting adherence was reported by some caregivers as a big challenge because most children intentionally do not want to take their drugs, since they feel they are not sick and their friends are not taking the drugs:

“...*the most painful of my experiences was the loss of this child's mother. From then, I found it very difficult to give the medication to the child, defaulting medication time…,*” said a 36-year-old female caregiver.

“*…and it is hard making him adhere to his drugs since he doesn't see his friends taking...,*” commented a 57-year-old father.

#### 3.2.2. Coping Strategies

From the analysis of the coping strategies, the following themes emerged as the most common strategies used by the caregivers in the study context: social support, spiritual support, and acceptance.

*(1) Social Support*. Findings from the qualitative arm regarding how respondents coped seem to support the results from the quantitative study. Sharing the problems faced by other friends caring for HIV-infected children (social support) was a theme that emerged as one of the commonly used coping strategies by the caregivers. This was exemplified by the following expressions:

“*...a friend of mine was almost giving up on caring for her child, I said to her, now that you know your child is suffering from HIV/AIDs, don't let her die, take care of her and you get blessings, and now the child is 8 years old and healthy...*,” said a 22-year-old mother.

“*...It was only one day that I and some other people caring for children living with HIV/AIDs underwent a training conducted by LUCITA in caring for these children, so this gave me the courage to continue caring for this child*,” said a 46-year-old male caregiver.

*(2) Spiritual Support.* Spiritual support also emerged as one of the common strategies used by the caregivers to cope with the burden of caregiving. A number of the respondents expressed attending prayer sessions, reading the Bible, and surrendering themselves to God as a way of getting relief from the burden of caregiving (spiritual support):

“*I have completely surrendered these children and myself to God. Without God, I wouldn't have persevered all this long together with these children. Therefore, I put God first and ART medication second*...,” one of the respondents asserted.

*(3) Acceptance*. Most caregivers expressed the fact that acceptance of the child's HIV status and the responsibility of caregiving did give them the strength and courage to cope with the challenges they encountered. This was exemplified by various statements as follows:

“*...we should continue being positive while caring for these children because it wasn't their will to be in this condition, to be born with HIV/AIDs. Let us look at them as if they are part of us, let us treat them equally with other children, and give them what other children also have,*” said a 43-year-old female caregiver.

“*...I advise other mothers to take care of their children with one heart and to also love them a lot. I advise other parents not to abandon their children who are on ARVs*,” said a 23-year-old mother.

“*My advice to other people caring for children living with HIV is that they should show them, love, be close to them and guide them when taking their medications and other information from the clinic concerning their health status, and give them food*,” commented a 36-year-old mother.

## 4. Discussion

There is a paucity of literature on caregiving and its associated burden among family caregivers of children living with HIV in low-resource settings. Consequently, there is a poor understanding of the care burden and how individuals and families respond to such a demand for chronic care. This study investigated the care burden among caregivers of HIV-infected children in a low-resource context and how the respondent population coped with the demands and the burden of caregiving. The study thus offers new insights and understanding of caregiver burden and coping strategies for paediatric HIV in resource-limited contexts.

### 4.1. Level of Care Burden

We found that the majority (65.6%) of the caregivers experienced mild-to-moderate burden, while 1.8% experienced severe burden. This finding contrasts with that reported by Ochigbo et al. in Nigeria, where the majority (76.4%) of the caregivers had no or minimal burden and only 16.4% had mild-to-moderate burden [28]. The level of care burden as found in the current study also differs from that reported among adults caring for people living with HIV/AIDS in Southern India, where 27.8% and 10.0% had mild-to-moderate and severe levels of burden, respectively [5]. The above variation could be attributed to differences in patient types and study contexts. We believe that the widespread experience of care burden in the context of our study could be attributed to the high level of poverty, illiteracy, and disruption of family support systems and could have important implications on the care of HIV-infected children, including poor retention, poor adherence, and low viral load suppression.

There were strong bearings of some specific items within the ZBI tool on the level of care burden experienced by the study participants. The highest scores were observed for the questions “Do you feel your health has suffered because of your involvement with your child?” (item 20), “Do you feel your child is dependent on you?” (item 8), and “Do you feel that your child seems to expect you to take care of him/her as if you were the only one he/she could depend on?” (item 14). This finding is similar to that previously reported by other authors [22, 29]. However, in contrast to previous reports, the response to the question “Are you afraid of what the future holds for your child?” (item 7) generated a low score. This finding is uniquely important and suggests a high level of conviction of hope among the respondents, as also expressed by respondents in the in-depth interview (qualitative finding) who accepted and confronted their situations, a factor shown to be important in handling the challenging situations of caregiving [30].

There was, however, a notably low mean score relating to the question “Do you feel that you don't have enough money to take care of your child in addition to the rest of your expenses?” (mean = 1.23), which was not in tandem with the expressions from the qualitative study where most of the respondents highlighted financial constraints as a major burden in caregiving. This is astounding and could partially be explained by the fact that respondents who participated in the in-depth interviews had “experienced it all in caring for an HIV-infected child” and reflects the role of exploratory studies in providing more perspectives to a context.

### 4.2. Factors Associated with Care Burden

The burden of caregiving was significantly influenced by certain caregivers' sociodemographic factors. Our results showed that caregivers aged 30–39 years experienced more burden than those in other age categories, which closely mirror those reported in a study by Rahmani et al. among caregivers of schizophrenic patients in Iran [11], suggesting a tendency to higher burden at an older age. Rutakumwa et al. in a previous study in Uganda contended that older persons encounter significant challenges in their caregiving role attributed to the high occurrence of poor health associated with advancing age, thus undermining their ability to optimally provide for children in their care [31]. The large number of caregivers aged ≥40 years (44.2%) in the current study should therefore be concerning. Our findings, however, contrast with those reported by Robson in Zimbabwe [32] and Lindsey et al. in Botswana [33], where young girls bore a high burden of caregiving with untoward consequences. Furthermore, our results suggest a significant association between divorce and an increase in the level of care burden experienced. This could be attributed to the role dynamics that these caregivers have to play in addition to caring for the HIV-infected child, compounded by the fact that divorce is a stressor in itself.

Caregivers with an average monthly income of less than 250,000 Uganda shillings (approximately $67) significantly experienced a higher burden compared to those with an average monthly income of ≥250,000 Shs, a finding that was also echoed by respondents in the qualitative study. This finding corroborates with that reported by Seng et al., which showed a lower care burden among caregivers who had fewer financial problems [22]. While Rahmani et al. reported a contrary finding of higher care burden among caregivers with perceived income adequacy [11], we believe the current finding may not be surprising, since caring for an HIV positive child requires financial resources to meet many of the child's needs. This is a situation reported by Kipp et al. in the pre-scale-up of ART in Uganda where all caregivers reported a deterioration of their economic status since becoming caregivers, with over half (59%) requesting direct financial assistance [34]. According to views from the qualitative arm of the study, financial constraints hindered access to basic needs such as food, clothing, and medical care, attesting to reports by other authors that lack of food is a significant predictor of caregiver burden among caregivers of people affected with HIV/AIDS [35, 36].

Furthermore, respondents caring for children who were attending school significantly experienced lower levels of care burden than those caring for children who were not in school. While the reason for this is not immediately obvious, we postulate that this could relate to the fact that, in this study, children who were not attending school were younger (median age = 7 years [IQR 4–7]) than those in school (median age = 9 years [IQR 7–9]) and therefore were more dependent on the caregiver. Furthermore, a higher proportion (36.4%) of children who were not attending school were reported to have been sickly compared to only 19.6% of children who were already at school, which, coupled with the younger age, is likely to have increased the level of care burden. This finding was also corroborated by respondents in the qualitative arm of the study where caregivers expressed difficulties caring for the children experiencing frequent illnesses and hospitalizations.

An important finding from the qualitative results highlighted the negative impact of stigma and discrimination on caregiving, in keeping with findings by Kalomo and Liao in rural Namibia, where HIV stigma experienced by the primary caregivers was associated with heightened caregiver burden [35]. Moore and Henry assert that intense stigma leads caregivers to feel overburdened by their caregiving demands [37, 38]. In this study, stigma made it difficult for caregivers to give the child's medications in the presence of other people, supporting a suggestion that intense HIV stigma often causes caregivers to keep their child's illness a secret, in turn putting their child's physical health at risk [37]. The finding of high levels of stigma and discrimination in our study is of great significance given the efforts put in by the ministry of health and its HIV implementing partners in addressing the problem of stigma and discrimination and should therefore call for more deliberate and bold change in approaches.

### 4.3. Coping Strategies and Related Caregivers' Sociodemographic Factors

Based on the original subscale, the three coping strategies with the highest median scores were Accepting Social Support, Seeking Spiritual Support, and Reframing, a finding similar to that reported by Guada (2012) in a study among African American families with a schizophrenic loved one [13]. By contrast, in a study among parents of children with cancer in Shiraz, Southern Iran, Spiritual Support ranked highest, followed by Seeking Help, Reframing, Passive Appraisal, and Social Support [14]. While these studies all used the same tool (F-COPES), they diverge on the disease spectrums studied. The high extent of use of social support among respondents in the current study as has also been reported by other authors [39, 40] is not unexpected given the sociocultural contexts where extended family and community systems form the basis of children's upbringing. Furthermore, the religious conviction as found in this study is in keeping with that reported by Osafo et al. in a qualitative study in Uganda where spirituality with high rates of religiousness was noted as a way of coping among caregivers [4]. This finding also bodes well with that found among caregivers of children with cancer in Iran [14] and is indeed in accord with the results of our qualitative findings where caregivers expressed turning to God for strength and hope, characterised by attending prayer sessions, reading the Bible, and believing that the disease will go away when they pray to God.

In this study, we also sought to investigate some more unique ways of coping among the sampled study population which were not suggested by the original subscales. We derived five coping factors from a principal component factor analysis of the F-COPES that best explained how the population in the context of this study responds to the demands and burden of caregiving for children living with HIV/AIDS. These factors could best be described as Internal Strength, Seeking Community Support, Information Seeking or Gathering, Acceptance, and Psychosocial Support. This finding has similarities with those reported among various population groups and disease contexts [13, 41, 42], with ingredients that can be considered as emotion- and problem-focused. These factors more reliably explained coping among the study population as shown by the improvement in the overall Cronbach's alpha coefficient from 0.68 to 0.71 and the marked improvement in the subscale coefficients compared to the original subscales.

The first factor, Internal Strength, demonstrates that caregivers relied on the inherent strengths and resilience within the family system to overcome the demands and burden of caregiving and presents a unique coping strategy that is not demonstrated in the original factor subscale. A similar finding has previously been reported by Guada who also contended that family interventions should emphasize a family's sense of its inherent capabilities for managing stress [13]. This is an important finding on which programs can leverage to support caregivers and families to explore and use their inherent strengths, where possible, as a first line of coping.

The sampled population also coped by seeking community support (the second factor), particularly from neighbours, while also taking a passive approach by believing that if they wait long, the problem will go away. Conceptually, this approach is similar to the original F-COPES subscale of Acquire & Accept Help [25]. The adoption of this strategy was significantly negatively correlated with the duration of the child's care, implying that the longer the child takes in care, the lesser the caregivers sought community support. Results from the qualitative data reaffirmed the role of this strategy in coping, where respondents shared their problems with persons similarly caring for children with HIV as a way of receiving support. This finding is of significance and demonstrates the importance of involving family members and the community in care [43], which reemphasizes the need to address the barriers of stigma, discrimination, and nondisclosure. These are barriers likely to hamper the beneficial roles of other family members and the community in coping.

The third factor centerd on Seeking Information from others as a means of dealing with stressors, particularly from friends and extended family members, in addition to seeking information and advice from persons in other families who have faced the same or similar problems. The use of this coping strategy was significantly positively correlated with being divorced or widowed but was less adopted as the caregiver drifts in the direction of being unemployed. Programs should therefore support a proactive strategy of providing useful information that aids coping, including information that addresses stigma and discrimination.

The fourth factor was labelled as Acceptance, an emotion-focused coping strategy that the family members utilize to cope [15, 42]. This is an important coping strategy and therefore a uniquely important finding not previously demonstrated in the original subscale. It has been argued that, by employing this strategy and accepting their difficult situations, families are better placed to redefine stressful events to make them more manageable [15]. Similarly, according to McCubbin et al., family members who use acceptance do not necessarily view their situation as negative but as a part of their everyday life, a fact which helps to reduce stress and improve the relationship with other family members [25].

The last factor, labelled as Psychosocial Support, is conceptually similar to the original subscale of Acquiring Social Support and has previously been reported [41, 44]. We found a positive correlation between average household income and the use of psychosocial support as a major coping strategy. This finding contrasts with that reported by Eaton et al. in a study among family members of hospitalized psychiatric patients who found no significant relationship between coping and the family's socioeconomic status [15]. This difference could be methodological and/or due to disease factors, since different diseases present unique challenges in caregiving.

This study has some limitations. One of the limitations is the relatively small sample size, which is likely to limit its external validity. However, this weakness was overcome by employing a mix-method design where findings from the qualitative study reinforced the quantitative data. Furthermore, being a cross-sectional study, it was not possible to establish any precise causal relationship between coping strategies, burden, and caregivers' sociodemographic factors.

Lastly, the preprint version of this manuscript has been submitted to Research Square and is available online [45].

## 5. Conclusions and Recommendations

This study shows that care burden is common among the caregivers of children living with HIV in the study context. Caregivers depend on both internal and external strengths for coping with the burden of caregiving. We recommend that appropriate health and social policies should be directed by programs supporting HIV care and treatment services to alleviate the caregiver burden in this and similar populations. Importantly, stakeholders involved in providing HIV/AIDS care, treatment, and support should (i) integrate livelihoods programs for families of children with HIV/AIDS and (ii) strengthen the social support systems like the mother/father support groups as a means for psychosocial support and dealing with stigma and discrimination.

## Figures and Tables

**Table 1:** Sociodemographic characteristics of study participants.

Characteristics	Frequency, *N* (%)
*Caregivers characteristics*	
Gender	
Male	28 (24.8)
Female	85 (75.2)
Age (years)	
18–29	21 (18.6)
30–39	42 (37.2)
≥40	50 (44.2)
Relation to child	
Mother	58 (51.3)
Father	20 (17.7)
Others	35 (31.0)
Highest level of education	
Primary	65 (57.5)
Secondary	28 (23.0)
Tertiary	11 (9.7)
No formal education	11 (9.7)
Occupation	
Formal employment	12 (10.6)
Self-employed	44 (38.9)
Peasant farmer	38 (33.6)
Unemployed	19 (16.8)
Marital status	
Single	54 (47.8)
Married	30 (26.5)
Divorced	10 (8.8)
Widowed	19 (16.8)
Children living with HIV/AIDS in household	
1 child	94 (83.2)
2 and more	19 (16.8)
Average monthly household income	
˂250,000	46 (40.7)
≥250,000	67 (59.3)

*Child characteristics*	
Gender	
Male	50 (44.2)
Female	63 (55.8)
Age (years)	
5 and below	17 (15.0)
Above 5	96 (85.0)
School attendance	
Yes	102 (90.3)
No	11 (9.7)
Duration in HIV care	
<10 years	96 (85.0)
≥10 years	17 (15.0)
Health condition since ART	
Healthy	89 (78.8)
Sickly	24 (21.2)

**Table 2:** Mean scores of caregivers' responses to care burden assessment questions (*n* = 113).

Item no.	ZBI care burden questions	Mean	SD	Variance
B1	Do you feel that your child needs more help than he/she needs?	3.07	1.03	1.07
B2	Do you feel that you don't have enough time for yourself because of the child?	1.48	1.30	1.70
B3	Do you feel stressed caring for the child and trying to meet other responsibilities for your family or work?	2.43	1.32	1.75
B4	Do you feel embarrassed about your child's condition?	0.75	1.17	1.37
B5	Do you feel angry when you are with your child?	0.76	1.10	1.20
B6	Do you feel that your child negatively affects your relationships with other family members or friends?	0.89	1.29	1.67
B7	Are you afraid of what the future holds for your child?	1.88	1.52	2.31
B8	Do you feel your child is dependent on you?	3.46	0.79	0.63
B9	Do you feel strained when you are around your child?	0.55	1.04	1.10
B10	Do you feel your health has suffered because of your involvement with your child?	1.33	1.31	1.72
B11	Do you feel that you don't have as much privacy as you would like because of your child?	0.86	1.22	1.50
B12	Do you feel that your social life has suffered because you are caring for your child?	0.59	1.13	1.28
B13	Do you feel uncomfortable about having friends because of your child?	0.53	1.17	1.36
B14	Do you feel that your child seems to expect you to take care of him/her as if you were the only one he/she could depend on?	3.38	0.96	0.92
B15	Do you feel that you don't have enough money to take care of your child in addition to the rest of your expenses?	1.23	1.45	2.09
B16	Do you feel that you will be unable to take care of your child much longer?	1.52	1.43	2.06
B17	Do you feel you have lost control of your life since your child's illness?	0.64	1.09	1.18
B18	Do you wish you could leave the care of your child to someone else?	1.12	1.36	1.84
B19	Do you feel uncertain about what to do about your child?	1.34	1.41	1.98
B20	Do you feel you should be doing more for your child?	3.58	0.69	0.48
B21	Do you feel you could do a better job of caring for your child?	3.02	1.16	1.34
B22	Overall, how burdened do you feel?	2.50	1.23	1.52

SD: standard deviation.

**Table 3:** Care burden scores and sociodemographic characteristics of caregivers of HIV-infected children in northern Uganda.

Variables	*n*	Mean ± SD	Statistics	*P* value
*Caregivers characteristics*				
Gender				
Male	28	34.54 ± 8.51	1.51^a^	0.135
Female	85	37.71 ± 10.00		
Age (years)				
18–29	21	31.76 ± 8.73	4.26^b^	**0.017** ^*∗*^
30–39	42	39.07 ± 10.70		
≥40	50	37.28 ± 8.56		
Relation to child				
Mother	58	37.76 ± 10.14	0.62^b^	0.541
Father	20	35.00 ± 9.29		
Others	35	36.63 ± 9.31		
Highest level of education				
Primary	65	37.45 ± 9.48	1.23^b^	0.304
Secondary	26	37.35 ± 9.04		
Tertiary	11	31.64 ± 12.81		
No formal education	11	38.09 ± 8.89		
Occupation				
Formal employment	12	38.83 ± 13.81	0.46^b^	0.710
Self-employed	44	37.73 ± 10.72		
Peasant farmer	38	35.92 ± 7.55		
Unemployed	19	35.84 ± 8.44		
Marital status				
Single	54	34.48 ± 8.32	3.53^b^	**0.017** ^*∗*^
Married	30	38.87 ± 8.62		
Divorced	10	44.00 ± 14.46		
Widowed	19	37.05 ± 10.34		
Children living with HIV/AIDS in HH				
1 child	94	36.23 ± 9.90	−1.68^a^	0.095
2 or more	19	40.32 ± 8.13		
Average monthly household income				
˂250, 000	46	39.24 ± 10.14	2.14^a^	**0.035** ^*∗*^
≥250, 000	67	35.33 ± 9.15		

*Child characterises*				
Age (years)				
5 and below	17	35.88 ± 10.00	−0.48^a^	0.635
Above 5	96	37.10 ± 9.70		
School attendance				
Yes	102	36.30 ± 9.30	−2.09^a^	**0.039** ^*∗*^
No	11	42.64 ± 11.99		
Duration in HIV care				
<10 years	96	37.03 ± 9.96	0.29^a^	0.774
≥10 years	17	36.29 ± 8.40		
Health condition since ART				
Healthy	89	36.13 ± 9.74	−1.67^a^	0.098
Sickly	24	39.83 ± 9.22		

^a^Independent *t*-test (df = 1); ^b^ANOVA (*F*); ^*∗*^*P* value is significant; HH = household.

**Table 4:** Descriptive statistics of caregivers' responses to the coping strategy questions (*n* = 113).

Item no.	Item	Mean	SD	Variance
C1	Sharing our difficulties with relatives	4.13	1.36	1.85
C2	Seeking encouragement and support from friends	4.12	1.29	1.67
C3	Knowing we have the power to solve major problems	2.80	1.55	2.41
C4	Seeking information and advice from persons in other families who have faced the same or similar problems.	4.13	1.27	1.62
C5	Seeking advice from relatives (grandparents, etc.)	4.14	1.24	1.53
C6	Seeking assistance from community agencies and programs designed to help families in our situation	2.01	1.47	2.17
C7	Knowing we have the strength within our family to solve our problems	2.74	1.51	2.28
C8	Receiving gifts and favours from neighbours	3.15	1.64	2.70
C9	Seeking information and advice from the family doctor	2.47	1.68	2.81
C10	Asking neighbours for favours and assistance	3.23	1.62	2.61
C11	Facing the problem head-on and trying to get the solution right away	3.71	1.55	2.41
C12	Watching T.V.	2.40	1.46	2.12
C13	Showing that we are strong	3.00	1.67	2.80
C14	Attending church services	4.91	0.34	0.12
C15	Accepting stressful events as a fact of life	4.46	1.04	1.07
C16	Sharing concerns with close friends	4.47	0.99	0.98
C17	Knowing luck plays a big part in how well we can solve family problems	1.81	1.17	1.37
C18	Accepting that difficulties occur unexpectedly	4.58	0.74	0.55
C19	Doing things with relatives (get-togethers, dinners, etc.)	4.34	1.09	1.19
C20	Seeking professional counselling and help for family difficulties	3.47	1.64	2.70
C21	Believing we can handle our problems	3.12	1.57	2.47
C22	Participating in church activities	4.80	0.70	0.49
C23	Defining the family problem more positively so that we do not become too discouraged	4.01	0.94	0.88
C24	Asking relatives how they feel about the problems we face	3.93	1.15	1.32
C25	Feeling that no matter what we do to prepare, we will have difficulty handling problems.	3.95	1.03	1.05
C26	Seeking advice from a minister	1.77	1.24	1.54
C27	Believing if we wait long enough, the problem will go away	3.91	1.31	1.71
C28	Sharing problems with neighbours	3.90	1.45	2.11
C29	Having faith in God	4.99	0.09	0.01

SD: standard deviation.

**Table 5:** Five-factor loading of items in the coping strategy (F-COPES) assessment scale (*n* = 113).

F-COPES items		Factor loadings				
		1	2	3	4	5
C1	Sharing our difficulties with relatives	0.102	−0.130	0.730^*∗*^	0.071	−0.016
C2	Seeking encouragement and support from friends	0.145	0.235	0.484^*∗*^	−0.342	−0.299
C3	Knowing we have the power to solve major problems	0.742^*∗*^	−0.009	−0.046	0.099	0.088
C4	Seeking information and advice from persons in other families who have faced the same or similar problems	0.129	0.272	0.426^*∗*^	−0.148	0.063
C5	Seeking advice from relatives (grandparents, etc.)	0.020	−0.129	0.671^*∗*^	0.177	0.185
C6	Seeking assistance from community agencies and programs designed to help families in our situation	0.084	−0.091	0.196	−0.142	0.442^*∗*^
C7	Knowing we have the strength within our family to solve our problems	0.812^*∗*^	0.033	−0.126	0.052	0.077
C8	Receiving gifts and favours from neighbours	0.175	0.831^*∗*^	0.026	0.016	0.092
C9	Seeking information and advice from the family doctor	0.265	0.100	0.094	−0.109	0.548^*∗*^
C10	Asking neighbours for favours and assistance	0.017	0.859^*∗*^	0.082	0.083	0.048
C11	Facing the problem head-on and trying to get the solution right away	0.497^*∗*^	0.409	0.089	0.230	0.034
C12	Watching T.V.	0.643^*∗*^	−0.017	0.322	−0.037	−0.066
C13	Showing that we are strong	0.737^*∗*^	−0.059	0.137	0.334	−0.226
C14	Attending church services	0.078	−0.022	−0.127	−0.266	0.069
C15	Accepting stressful events as a fact of life	−0.083	0.305	−0.063	0.418^*∗*^	−0.580
C16	Sharing concerns with close friends	−0.215	0.305	0.541^*∗*^	−0.197	−0.277
C17	Knowing luck plays a big part in how well we can solve family problems	−0.120	−0.085	−0.107	−0.713	0.145
C18	Accepting that difficulties occur unexpectedly	−0.007	0.245	0.065	0.120	−0.717
C19	Doing things with relatives (get-togethers, dinners, etc.)	0.216	0.143	0.535^*∗*^	0.142	0.111
C20	Seeking professional counselling and help for family difficulties	−0.104	0.074	0.017	−0.125	0.551^*∗*^
C21	Believing we can handle our problems	0.672^*∗*^	−0.075	0.049	−0.120	0.072
C22	Participating in church activities	−0.003	−0.168	0.199	0.454^*∗*^	0.029
C23	Defining the family problem more positively so that we do not become too discouraged	0.378	0.120	−0.051	0.632^*∗*^	−0.103
C24	Asking relatives how they feel about the problems we face	−0.135	0.141	0.526^*∗*^	0.244	0.010
C25	Feeling that no matter what we do to prepare, we will have difficulty handling problems	0.105	0.170	−0.115	0.592^*∗*^	−0.025
C26	Seeking advice from a minister	−0.062	0.110	−0.103	0.271	0.526^*∗*^
C27	Believing if we wait long enough, the problem will go away	−0.220	0.480^*∗*^	−0.046	0.311	−0.107
C28	Sharing problems with neighbours	−0.251	0.614^*∗*^	0.272	−0.025	−0.216
C29	Having faith in God	0.183	−0.089	0.036	−0.089	−0.214

**Table 6:** Correlation coefficients and standardized beta weights between the five coping subscales and caregiver and child sociodemographic characteristics (*n* = 113).

	Factor 1		Factor 2		Factor 3		Factor 4		Factor 5	
	*R*	*b*	*r*	*b*	*r*	*b*	*r*	*b*	*r*	*b*
Gender	0.052	−0.101	−0.115	−0.034	−0.128	−0.236^*∗*^	0.017	−0.033	−0.149	−0.103
Age	−0.118	−0.129	0.055	0.085	0.105	−0.021	0.041	0.049	−0.036	0.029
Relation	−0.103	−0.024	−0.103	−0.095	0.061	0.063	−0.025	−0.050	−0.009	−0.040
Education	0.044	−0.039	−0.180	−0.031	0.098	0.125	0.025	0.025	0.158	0.063
Marital status	0.155	0.160	−0.152	−0.117	0.258^*∗∗*^	0.285^*∗*^	−0.029	−0.103	−0.020	0.000
Occupation	−0.311^*∗∗*^	−0.256	0.176^*∗*^	0.069	−0.223^*∗*^	−0.196^*∗*^	0.035	−0.003	−0.129	−0.114
Income	0.047	0.054	−0.149	−0.153	−0.175	−0.202	0.003	0.015	0.360^*∗∗*^	0.314^*∗*^
No. of HIV+ children	−0.055	0.003	0.152	0.209	0.029	0.028	0.125	0.124	−0.046	0.098
Child's age	−0.145	−0.262	−0.054^*∗*^	0.128	0.073	0.037	−0.172	−0.264^*∗*^	0.083	0.024
Schooling	0.084	0.058	−0.014	−0.049	0.092	0.084	−0.079	−0.105	−0.232^*∗*^	−0.143
Duration in care	0.098	0.220	−0.254^*∗∗*^	−0.295^*∗*^	0.079	0.003	0.003	0.145	0.034	0.015
Child's health	0.091	0.036	−0.113	−0.058	−0.009	−0.070	−0.037	−0.029	0.061	0.080
Adj *R*^*2*^		0.075		0.075		0.113		−0.042		0.082

Factor 1: Internal Strength; Factor 2: Seeking Community Support; Factor 3: Information Seeking; Factor 4: Acceptance; Factor 5: Psychosocial Support.  ^*∗*^*P* < 0.05;  ^*∗∗*^*P* < 0.01; *r* = Pearson's coefficients; *b* = standardized beta coefficients.

## Data Availability

The datasets used and/or analysed during the current study are available from the corresponding author upon request.

## Abbreviations

AIDS: Acquired immune deficiency syndrome
ART: Antiretroviral therapy
HIV: Human immunodeficiency virus
LRRH: Lira Regional Referral Hospital.
